# JAK inhibitors in livedoid vasculopathy associated with thrombophilia and refractory to anticoagulation: report and literature review^؆^

**DOI:** 10.1016/j.abd.2026.501324

**Published:** 2026-03-23

**Authors:** Giulia Jardim Criado, Paulo Ricardo Criado, Daniel Lorenzini, Hélio Amante Miot

**Affiliations:** a Faculdade de Ciências Médicas de Santos, Centro Universitário Lusíada, Santos, SP, Brazil; b Faculty of Medicine, Centro Universitário do ABC, Santo André, SP, Brazil; c Dermatology Service, Santa Casa de Misericórdia de Porto Alegre, Porto Alegre, RS, Brazil; d Department of Infectology, Dermatology, Diagnostic Imaging and Radiotherapy, Faculdade de Medicina, Universidade Estadual Paulista, Botucatu, SP, Brazil

Dear Editor,

Livedoid vasculopathy (LV) is a rare, chronic, and recurrent pauci-inflammatory thrombo-occlusive condition characterized by painful ulcers and livedoid changes in the lower limbs, often exacerbated by heat exposure.^1^

There are no controlled trials on therapy in cases refractory to anticoagulation, posing a challenge to dermatologists. This report describes a patient with LV and thrombophilia whose anticoagulation did not control the disease; however, therapy with Janus Kinase inhibitors (JAKi) induced prolonged remission.

A 35-year-old Caucasian man presented with violaceous macules on his feet and ankles that progressed to painful reticulated ulcers with a seasonal pattern of worsening over the past two years. After identifying a heterozygous factor V (Leiden) mutation, anti-stasis measures and acetylsalicylic acid (100 mg/day) were initiated, controlling the disease for 11 months; however, with a severe relapse the following summer. Acetylsalicylic acid was then replaced with rivaroxaban (20 mg/day), maintaining clinical control for two years, with a new relapse.

Abrocitinib (200 mg/day) was initiated, leading to complete clinical remission in two months (Fig. 1A). Abrocitinib was replaced with baricitinib (4 mg/day) due to a change in health insurance coverage, and rivaroxaban was discontinued without loss of efficacy, maintaining a complete response in the ten-month follow-up (Fig. 1B).

**Fig. 1:**
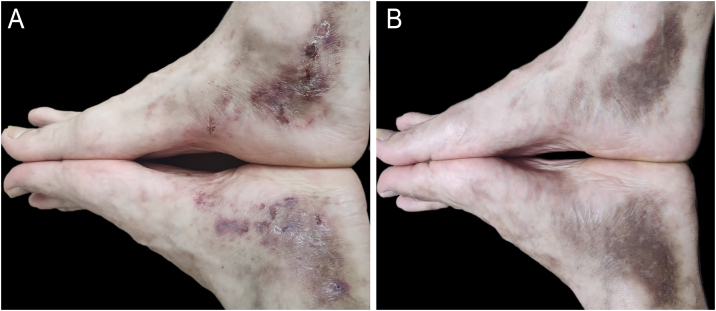
Livedoid vasculopathy. (A) Typical ulcerated and purpuric lesions on the ankles and feet, with progressive resolution of the LV after one month of abrocitinib use. (B) Follow-up after six months, showing complete remission with baricitinib use.

Table 1 shows 16 recovered LV cases from the literature review up to June 2025 (PubME/Medline and LILACS), which were treated with JAK inhibitors. In 13 of them, no thrombophilias were detected, and in three others, this was not reported by the authors.

**Table 1:** Cases of livedoid vasculopathy in adults and children with reported success using oral JAK inhibitors.

Report	Sex	Age	Previous treatments	JAK Inhibitor	Remission / Follow-up
1^a^	Female	48 years	Colchicine, methotrexate, dapsone, corticosteroids, heparin, rivaroxaban, acetylsalicylic acid, intravenous immunoglobulin	Baricitinib 4 mg/d	3 years
2^a^	Female	43 years	Dapsone, rivaroxaban, acetylsalicylic acid, intravenous immunoglobulin	Upadacitinib 15 mg/d	6 months
3^b^	Female	32 years	Acetylsalicylic acid, hydroxychloroquine, pentoxifylline, prednisone	Tofacitinib 10 mg/d	12 months
4^c^	Female	14 years	Rivaroxaban	Baricitinib 4 mg/d	9 months
5^d^	Male	17 years	Colchicine, thalidomide, dipyridamole, rivaroxaban, methylprednisolone, acetylsalicylic acid	Tofacitinib 10 mg/d	13 months
6^e^	Female	14 years	Anticoagulants (?), herbal medicines	Baricitinib 4 mg/d	9 months
7^f^	Male	8 years	Prednisone, herbal medicines	Baricitinib 2 mg/d	18 months
8^f^	Male	26 years	Thalidomide, cetirizine, prednisone	Baricitinib 2 mg/d	4 months
9^f^	Female	26 years	Prednisone, rivaroxaban, thalidomide, acetylsalicylic acid	Baricitinib 2 mg/d	4 months
8^g^	Female	26 years	Prednisone, thalidomide, diosmin, acetylsalicylic acid	Baricitinib 4 mg/d	12 months
9^h^	Female	43 years	Prednisone, methotrexate, warfarin	Tofacitinib 10 mg/d	12 months
10^i^	Female	31 years	Not mentioned (multiple)	Abrocitinib 100 mg/d	3 months
11^j^	Female	48 years	Pentoxifylline, nifedipine, acetylsalicylic acid, corticosteroids, etanercept	Baricitinib 4 mg/d	4 months
12^j^	Female	36 years	Corticosteroids, thalidomide, rivaroxaban, acetylsalicylic acid, enoxaparin	Baricitinib 2 mg/d	16 weeks
13^j^	Male	17 years	Corticosteroids, thalidomide, rivaroxaban, acetylsalicylic acid, enoxaparin	Baricitinib 2 mg/d	10 weeks
14^j^	Female	26 years	Corticosteroids, thalidomide	Baricitinib 2 mg/d	14 weeks
15^j^	Female	18 years	Prednisone, herbal medicines	Baricitinib 2 mg/d	16 weeks
16^j^	Female	12 years	Prednisone, herbal medicines	Baricitinib 2 mg/d	16 weeks

*The references for the reports cited in the table are listed as Supplementary Material.

Long-term remission of LV refractory to anticoagulation reinforces the potential of JAKi in modulating inflammatory and immunological pathways in thrombo-inflammatory conditions such as LV, which is typically associated with hypercoagulable states.^1^, ^2^ Thrombosis and inflammation are feedback responses in LV. Fig. 2 illustrates the proposed mechanisms of involvement of the JAK/STAT pathway in the pathogenesis of LV, with the Venn diagram in the upper left corner showing the multicausality of livedoid vasculopathy. The black arrows indicate pathophysiological events of thrombosing vasculopathy with ischemia and tissue damage, which contribute to the activation of innate immunity and mild local inflammation, and may be mediated by the JAK/STAT system; the green arrows indicate alterations in innate immunity triggered by ischemia, with the oxidation of Lipoprotein(a) [Lp(a)], which are related to interferon production and the JAK/STAT system activation; the red arrows indicate the consequences of JAK/STAT system activation, with increased P-selectin, acting to reduce fibrinolysis, increased thrombosis, and the generation of inflammatory mediators such as inducible Nitric Oxide Synthase (iNOS) and Cyclooxygenase-2 (COX-2), causing perilesional erythema and local pain.

**Fig. 2:**
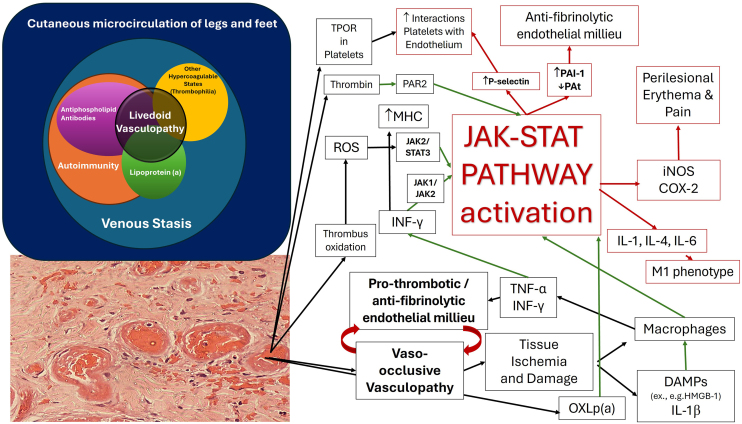
Proposed mechanisms of JAK/STAT pathway involvement in the pathogenesis of livedoid vasculopathy, promoting a prothrombotic and antifibrinolytic environment in the cutaneous environment. COX2, Cyclooxygenase 2; DAMPs, Damage-Associated Molecular Patterns; HMGB1, High-Mobility Group B Protein 1; iNOS, Inducible Nitric Oxide Synthase; IL, Interleukin; IL-1β, Interleukin 1 beta; JAK-STAT, Janus Kinase-Signal Transducer and Transcription Activator; PAI 1, Plasminogen Activator Inhibitor type 1; MHC, Major Histocompatibility Complex; PAR2, Protease-Activated Receptor type 2; ROS, Reactive Oxygen Species; tPA, Tissue Plasminogen Activator; TNF-α, Tumor Necrosis Factor alpha; TPO-R, Thrombopoietin Receptor.

IL-2 and its soluble receptor are elevated in the serum of patients with LV. Increased tissue expression of platelet P-selectin was observed, suggesting platelet and lymphocyte activation in patients with VL.^3^ Furthermore, serum lipoprotein (a) [Lp(a)] and antiphospholipid antibodies (aPL) play a central role in the pathogenesis of LV. Lp(a) shares structural homology with plasminogen, interrupting fibrinolysis and promoting fibrin deposition in small-caliber vessels.^4^ Additionally, aPL activate the endothelium and the complement system, exacerbating microvascular thrombosis.^5^

Lp(a) also induces ICAM-1 expression in the endothelium and reduces TGF-β availability. It also promotes monocyte adhesion and transendothelial migration by stimulating the expression of monocyte chemoattractant protein-1 and chemokine I-309. This process facilitates the differentiation of pro-inflammatory M1-type macrophages, leading to the activation of helper T cells and NK cells. Lp(a) also stimulates the local secretion of IL-1β, TNFα, IL-8, IL-6, VCAM-1, and E-selectin in endothelial cells.^6^

In primary antiphospholipid syndrome, aPL antibodies can trigger the release of type I interferons (α/β) via the plasmacytoid dendritic cell pathway, via activation of TLR7 and TLR9. Type I interferons can induce endothelial dysfunction and increase the expression of pro-thrombotic molecules, promoting fibrin and C5b-9 deposition, which contributes to the thrombosis characteristic of the disease.^7^, ^8^ Additionally, type I interferons activate STAT1, STAT2, and STAT4 via JAK1 and TYK2 signaling.^9^

During thrombosis, activation of cytokine receptors on platelets, such as the Thrombopoietin Receptor (TPO-R), triggers the JAK-STAT pathway, intensifying platelet interaction and promoting thrombus formation. Thrombin activates Protease-Activated Receptor 2 (PAR2) on endothelial cells, modulating the JAK-STAT pathway responsible for stimulating or inhibiting thrombus formation or lysis, respectively, via tissue plasminogen activator (tPA) and plasminogen activator inhibitor type 1 (PAI-1). In addition, IL-4 and IL-6 polarize dermal macrophages to the M1 phenotype via the JAK-STAT pathway, prolonging tissue damage and compromising the M2 phenotype, which is necessary for tissue remodeling.^10^

Thus, pro-inflammatory cytokines involved in LV, driven by aFL, Lp(a) expression, and JAK-STAT pathway activation suggest a rationale for the use of JAKi in this clinical context based on: (i) JAK1-2 are intracellular signaling pathways of cIFNα, IFNβ, IFNγ, IL-2, and IL-6; (ii) JAKi reduce endothelial activation and the release of pro-inflammatory mediators; (iii) IL-6 inhibition reduces tissue factor expression and modulates PAI levels, helping to restore the balance between coagulation and fibrinolysis; (iv) JAKi effectively reduce vascular adhesion molecules such as ICAM-1 and suppress cytokine production, especially TNFα and IL-1β induced by oxidized LDL.^10^ A recent systematic review indicated JAKi as a safe and effective alternative in the treatment of refractory LV.^11^

In conclusion, this report reiterates evidence of JAKi as a promising therapeutic option in LV, especially when refractory to anticoagulation, even in the presence of thrombophilia.

## ORCID ID

Giulia Jardim Criado: 0009-0006-7360-6267

Paulo Ricardo Criado: 0000-0001-9785-6099

Daniel Lorenzini: 0000-0002-6850-5799

Hélio Amante Miot: 0000-0002-2596-9294

## Financial support

CNPq (306358/2022-0 e 305330/2022-5) – Hélio Miot and Paulo Criado are research fellows of CNPq.

## Authors' contributions

Giulia Jardim Criado: Collection of data; design and planning of the study; analysis and interpretation of data; drafting and editing of the manuscript; critical review of the literature; critical review of the manuscript; approval of the final version of the manuscript.

Daniel Lorenzini: Collection of data; design and planning of the study; analysis and interpretation of data; drafting and editing of the manuscript; critical review of the literature; critical review of the manuscript; approval of the final version of the manuscript.

Paulo Ricardo Criado: Collection of data; design and planning of the study; analysis and interpretation of data; drafting and editing of the manuscript; critical review of the literature; critical review of the manuscript; approval of the final version of the manuscript.

Hélio Amante Miot: Design and planning of the study; analysis and interpretation of data; drafting and editing of the manuscript; critical review of the literature; critical review of the manuscript; approval of the final version of the manuscript.

## Research data availability

Does not apply.

## Conflicts of interest

Giulia Criado: None declared.

Dr. Paulo Criado: Advisory board ‒ Pfizer, Galderma, Takeda, Hypera, Novartis, Sanofi; Clinical Research ‒ Pfizer, Novartis, Sanofi, Amgen and Lilly; Speaker: Pfizer, AbbVie, Sanofi-Genzyme, Hypera, Takeda, Novartis.

Dr. Daniel Lorenzini: Advisory board ‒ Pfizer, Galderma Clinical Research ‒ Pfizer, Novartis, Sanofi, Amgen and Lilly; Speaker.

Dr. Hélio Miot: Advisory Board – Johnson & Johnson, L’Oréal, Theraskin, Sanofi and Pfizer; Clinical Research ‒ AbbVie, Galderma, Pierre-Fabre, and Merz.
