# Supplementary material for: JAK inhibitors in livedoid vasculopathy associated with thrombophilia and refractory to anticoagulation: report and literature review
Source: An Bras Dermatol. 2026 Mar 23;101(2):501324. doi: 10.1016/j.abd.2026.501324 (PMC13049387; doi:10.1016/j.abd.2026.501324)
Supplement: Supplementary file 1 [file mmc1.docx]

ABD-D-25-00402_Supplementary Material

*** Supplementary Material**

Bibliographic references from Table 1.

^a^ Delpuech A, Tournier E, Sohier P, Dupin N, Challamel C, Altandi S, et al. Livedoid Vasculopathy Successfully Treated with JAK Inhibitors. JEADV Clinical Practice. 2025. (ahead of print): doi: 10.1002/jvc2.593.

^b^ Mansouri P, Mozafari N. Effective Treatment of Livedoid Vasculopathy with Oral Tofacitinib. Clin Case Rep. 2025;13:e70536.

^c^ Liu C, Jin YY, Han X, Huang H, Bao S, Xu X, et al. Baricitinib Successfully Treated a Teenager with Refractory Livedoid Vasculopathy: A Case Report and Literature Review. J Inflamm Res. 2025;18:1471-7.

^d^ Jia E, Yan G, Xiao M, Geng H, Wei J, Zhang J. Refractory ulcerations associated with livedoid vasculopathy successfully treated with tofacitinib. Dermatol Ther. 2020;33:e14470.

^e^ Zhang H, Chen J, Wu N, Chen H, Liu Y. Refractory livedoid vasculopathy in a child successfully treated with baricitinib. Dermatol Ther. 2022;35:e15659.

^f^ Song X, Tu P. Treatment of Livedoid Vasculopathy with Baricitinib. JAMA Dermatol. 2022;158:587-9.

^g^ Xiao Y, Wang Y, Gu Y, Xia D, Li W. Refractory livedoid vasculopathy successfully treated with baricitinib. Int J Dermatol. 2023;62:1204-5.

^h^ Rudrakar R, Kumar A. Livedoid vasculopathy: successful treatment with tofacitinib. Indian J Rheumatol. 2023;18:344-5.

^i^ Chen P, Liang J, Li C, Li Q, Liu W, Zhu J, et al. Abrocitinib as a Novel Treatment for Multiple Skin Disorders: 3 Case Reports and a Scoping Review. Clin Cosmet Investig Dermatol. 2024;17:35-40.

^j^ Peñuelas Leal R, Grau Echevarría A, Martínez Domenech Á, Hoyos CL, Ninet VZ, Carazo JLS, et al. Refractory livedoid vasculopathy successfully treated with baricitinib. Int J Dermatol. 2023;62(11):e608–e10.
